# Analyzing Sensor-Based Individual and Population Behavior Patterns via Inverse Reinforcement Learning

**DOI:** 10.3390/s20185207

**Published:** 2020-09-12

**Authors:** Beiyu Lin, Diane J. Cook

**Affiliations:** 1 Department of Computer Science, the University of Texas Rio Grande Valley, Edinburg, TX 78539, USA; beiyu.lin@utrgv.edu; 2 School of Electrical Engineering and Computer Science, Washington State University, Pullman, WA 99163, USA

**Keywords:** smart homes, ambient sensors, activity recognition, behavior analysis, inverse reinforcement learning

## Abstract

Digital markers of behavior can be continuously created, in everyday settings, using time series data collected by ambient sensors. The goal of this work was to perform individual- and population-level behavior analysis from such time series sensor data. In this paper, we introduce a novel algorithm—Resident Relative Entropy-Inverse Reinforcement Learning (RRE-IRL)—to perform an analysis of a single smart home resident or a group of residents, using inverse reinforcement learning. By employing this method, we learnt an individual’s behavioral routine preferences. We then analyzed daily routines for an individual and for eight smart home residents grouped by health diagnoses. We observed that the behavioral routine preferences changed over time. Specifically, the probability that the observed behavior was the same at the beginning of data collection as it was at the end (months later) was lower for residents experiencing cognitive decline than for cognitively healthy residents. When comparing aggregated behavior between groups of residents from the two diagnosis groups, the behavioral difference was even greater. Furthermore, the behavior preferences were used by a random forest classifier to predict a resident’s cognitive health diagnosis, with an accuracy of 0.84.

## 1. Introduction

Humans have long sought to understand their own behavior and the influence of a person’s behavior on themselves, those nearby, and society. In response to this need, theories have arisen from psychology, sociology, and anthropology to explain the complexities of behavior and the factors that drive it [1,2,3]. Such theories have relied on self-reporting. However, these sources of information are prone to error due to retrospective memory limitations [4] and unintended experimenter bias [5]. Over the past decade, sensors have become small, low-power, low-cost, high-capacity, and easily integrated into everyday settings [6,7,8,9,10]. As a result, we now have the ability to perform automated behavior monitoring and analysis.

### 1.1. Background

The long-term goal of this research is to perform population-level analyses of behavior from ambient sensor data. By modeling the behavior of an individual or a group from sensor data, we introduce a method to quantitatively link the health status with behavior, providing a foundation for automated health assessment and the design of behavior-driven interventions. Assessing and promoting health are challenging tasks at the best of times because health care providers must make decisions based on a typical 20 min visit with a patient [11], aided by complex, often-noisy laboratory tests. The ability to provide real-time, accurate assessments is particularly timely because, as the population ages, older adults will likely outnumber children for the first time in US history [12], creating a discrepancy between the number of persons needing care and those capable of providing it [12,13,14,15,16]. As a result, chronic illness rates and healthcare expenditures are both increasing [15,16]. The early detection of cognitive health changes with the close monitoring of chronic conditions has been identified as a national priority [17,18] because this supports more effective treatment and significantly improves the quality of care while reducing health care costs [19,20]. However, clinic-based assessment is infeasible for many who live in remote areas or remain in their homes due to COVID-19 or travel restrictions. Additionally, spending a few minutes in a doctor’s office or clinic does not provide the same insights that can be gleaned from the continuous monitoring of relevant behavioral and physiological data.

Because a strong relationship exists between behavioral habits and a person’s health status, we hypothesize that a health diagnosis can be inferred based on sensor-observed behavior patterns. Furthermore, we postulate that behavior patterns can be gleaned from machine learning methods and that these same methods can be used to differentiate behavior between points in time for a single individual, between multiple individuals, and between groups of people representing diverse health diagnosis groups.

To model human behavior from ambient sensor data, we propose an approach that is based on inverse reinforcement learning (IRL). IRL is a machine learning technique that mimics observed behavior. In this paper, our proposed algorithm—Resident Relative Entropy-Inverse Reinforcement Learning (RRE-IRL)—mimics the behavior of a smart home resident based on sensor-observed navigation patterns within a home environment. Unlike other IRL research, which learns from imitation to recreate expert actions, we learn from mimicry to capture and analyze a model of human behavior. We describe a method for modeling navigation patterns as a Markov decision process. Next, we learn a behavior strategy, or policy, that is consistent with the observed movement patterns. Finally, we use the created framework to analyze behavior patterns and behavioral differences for residents living in eight actual smart homes.

### 1.2. Related Work

With the increasing ability to collect a massive amount of sensor data on subjects in an unobtrusive manner, we can now design data mining methods to better understand human behavior. Researchers employ computational techniques to understand the relationship between behavioral habits and one’s health status [21,22,23,24,25]. Previous work has also considered the quantification of behavioral change while a person is experiencing health events, although this was only considered for individuals and not for groups [26]. Previously, studies have constructed formal models of human dynamics from digitally-derived information. As examples, formal methods have modeled a single spatial or temporal feature, such as the inter-arrival time of two successive events (e.g., the time delay between two occurrences of the same activity), as Poisson processes and heavy-tailed distributions [27,28,29,30,31,32]. Another prior approach analyzes spatio-temporal human processes with Markov models [33,34,35,36].

When data are combined from multiple sources, such as multiple heterogeneous features, a challenge arises in creating a model that can include all available information. For our analysis, we wanted to construct a model that combines resident temporal and spatial information. The selected model also had to be able to learn behavior preferences and distinguish behavior strategies between individuals and population groups.

One data-driven approach to behavior modeling that meets these constraints is inverse reinforcement learning (IRL) [37]. IRL is effective at imitating the observed actions of another agent or person. IRL learns a reward that reflects the observed agent’s actions, and traditional reinforcement learning can then formulate a policy of actions that are consistent with that reward. The end result is an artificial agent that tackles a problem in a manner similar to the observed person. This person may be an expert in the problem domain or, as in our work, an individual whose behavior we want to better understand. Because researchers realize the value of IRL for training artificial agents in solving difficult problems, researchers have introduced several IRL variations, including Maximum Entropy [38,39], Relative Entropy [40,41], and Bayesian IRL [42,43]. Existing techniques can be broadly categorized into model-based approaches [39] and model-free approaches [41,44]. The former assume that prior knowledge of system dynamics is provided, while the latter work on the assumption that such prior information is unknown. For many real-world problems, such as smart home behavior, reliable priors are not provided. Therefore, we adopted a model-free approach. This approach is common for complex real-world applications, including analyzing taxi driver movement patterns [45] and routine commuting patterns for other workers [46], assessing a person’s mental health [46], and modeling clinical rehabilitation stimulation motions [43]. 

## 2. Materials and Methods

The goal of this research was to analyze and compare human behavior from ambient sensor data. Our study offers a unique contribution to IRL by analyzing ambient sensor-based human routine behavior in home settings using inverse reinforcement learning. To describe our methods, we first formalize the IRL problem, and then describe the smart home sensor data collection environment and characterize the IRL strategy for this problem. The culmination of this process is a new algorithm—RRE-IRL—which we used to analyze smart home behavior patterns.

### 2.1. Inverse Reinforcement Learning

Designing a reinforcement learning or inverse reinforcement learning solution to a problem relies on first defining a representation for the problem’s state space and a set of possible actions. To accomplish this, we modeled a smart home resident’s sequential decision-making space as a Markov Decision Process. Based on this model, we learnt a resident’s behavioral routine via relative entropy inverse reinforcement learning.

#### 2.1.1. Markov Decision Process

A Markov Decision Process (MDP) is a discrete-time control process in which the outcomes of actions are probabilistic. An MDP can be characterized by the tuple (*S, A, T, γ, D, R*) [47]. In this characterization, *S* represents the set of possible states; *A* is a set of actions the agent can perform; and *T* represents the set of state transition probabilities, where *T = {P(s_j_|s_i_,a_i_)}* is the probability that the agent will be in state *s_j_* after executing action *a_i_*, while in state *s_i_*, ∀*s_j_, s_i_ ∈ S, a_i_ ∈ A*. Additionally, R(*s_i_, a_i_*) is a function specifying the reward that an agent will receive for executing action *a_i_* from state *s_i_* [48]. Because the reward may accumulate over multiple actions in a sequence, *γ ∈ [0, 1]* represents the discount factor that is applied to the reward, thus avoiding collecting arbitrarily large rewards for arbitrarily-long sequences of actions. Finally, D represents a distribution over possible start states, *s_0_*. For convenience, we let MDP\R denote an MDP that does not utilize a reward function, or a tuple (*S, A, T, D).*

#### 2.1.2. Reinforcement Learning

An MDP assumes that an agent can navigate to any destination state based on a sequence of actions that is suggested by a corresponding strategy. This strategy, or policy *π*, guides an agent in selecting an action from any given state. Given the large space of potential policies, an agent ideally wants to select an optimal policy, which is one that maximizes the total discounted rewards of executing the policy. Because randomness is inherent in an MDP (e.g., the initial state distribution and the transition probabilities), maximizing the accumulated rewards is adjusted to instead maximize the expected accumulated rewards. The goal of reinforcement learning (RL) is to find an optimal policy, *π*, that maximizes the expected sum of discounted rewards [49], as shown in Equation (1).

(1) $$\pi =\arg\max_{\pi }\;E\left[\overset{\infty }{\underset{i=0}{\sum }}\gamma^{i}R\left(s_{i},a_{i}\right)|\pi \right],\forall s_{i}\in S,{\;a}_{i}\in A$$

#### 2.1.3. RRE-IRL 

In the case of an MDP\R problem, one RL challenge is to find an optimal policy when the rewards are unknown. This is where inverse reinforcement learning plays an important role, because IRL extracts a reward function given the observations of an agent executing actions [50]. Often, IRL learns a reward function from observing an agent perform a plan. If the agent is an expert in the problem domain, this process allows IRL to learn the reward that is consistent with the expert’s reward function. RL can learn a policy consistent with the reward, thus allowing the agent to imitate the expert’s strategy. In this way, an automated agent can imitate the strategy of the expert. For our proposed work, we consider learning a reward function of a smart home resident. The goal is not to optimize the performance of actions within a smart home by imitating an expert, but to model and analyze the reward function that drives behavior [51] for a particular smart home resident.

**Figure 1:**
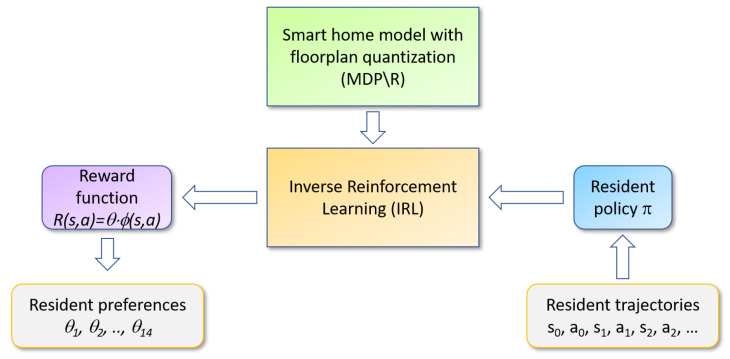
Resident Relative Entropy-Inverse Reinforcement Learning (RRE-IRL) analysis of smart home sensor data.

Figure 1 illustrates our analysis process. Our proposed RRE-IRL algorithm accepts a set of trajectories observed over time for a smart home resident as the input. The trajectories are consistent with the resident’s behavior patterns, or policy *π*, that govern their choice of actions for each state (place and time). Inverse reinforcement learning processes this information, together with the floorplan MDP\R, to generate a corresponding reward function. Finally, the reward function is decomposed into constituent pieces that can be used to analyze a person’s behavioral strategies and distinguish the behavior policies between individuals and groups.

We assumed that the “true” reward function, *R*, exists, and that it can be expressed as a linear combination of a feature vector *ϕ* with a corresponding weight vector, *θ*, $R\left(s_{i},a_{i}\right)=\theta \cdot \phi \left(s_{i},a_{i}\right)$, for $\forall \left(s_{i},a_{i}\right)\in S\times A$. For our study, vector *ϕ* indicates desiderata of a resident when spending time in their home, such as whether they prefer staying in the bedroom or office room, whether they frequently visit the kitchen sink, and so on. The unknown vector *θ* specifies the relative weight, or preference, between these desiderata. Learning the reward function can be specified as an optimization problem. Specifically, Equation (1) can be rewritten as shown in Equation (2).

(2) $$\begin{array}{c} \pi =\arg max_{\pi }\;E\left[\overset{\infty }{\underset{i=0}{\sum }}\gamma^{i}\theta \cdot \phi \left(s_{i},a_{i}\right)|\pi \right]\\ =\arg max_{\pi }\theta \cdot E\left[\overset{\infty }{\underset{i=0}{\sum }}\gamma^{i}\cdot \phi \left(s_{i},a_{i}\right)|\pi \right],\forall s_{i}\in S,\;a_{i}\in A \end{array}$$

Given Equation (2), we can define μ(π) as the feature expectations, or the vector of expected discounted feature values for a policy π. The vector can be expressed as shown in Equation (3).

(3) $$\mu \left(\pi \right)=E\left[\overset{\infty }{\underset{i=0}{\sum }}\gamma^{i}\cdot \phi \left(s_{i},a_{i}\right)|\pi \right]$$

When we substitute μ(π) back into Equation (2), we yield Equation (4).

(4) $$\pi =\arg max_{\pi }\;\theta \cdot \mu \left(\pi \right)$$

Here, the feature expectation, μ(π), determines the expected sum of rewards. That is, to find an optimal policy in an MDP\R problem where rewards are unknown, we find the maximum value of feature expectations instead of the maximum value of the expected sum of rewards.

### 2.2. CASAS Smart Home

**Figure 2:**
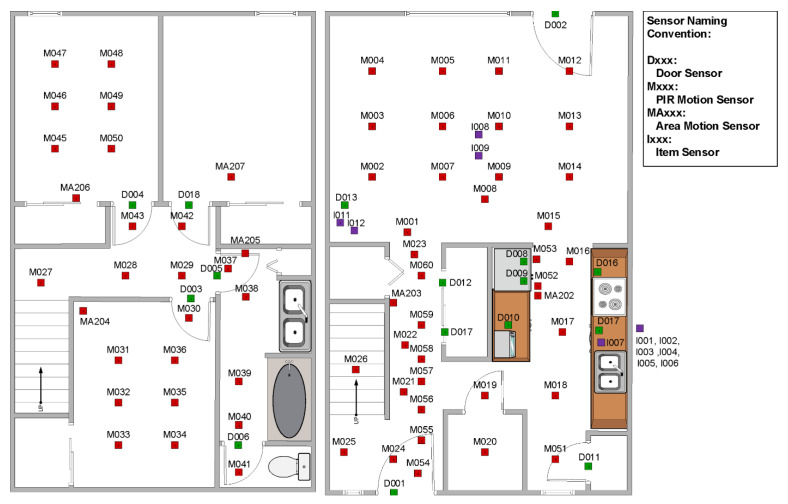
Floor plan and sensor locations for an on-campus smart home testbed. Motion/light sensor locations are indicated with red squares and door/temperature sensor locations are indicated with green squares. Purple squares indicate the locations of items that are tagged with additional sensors.

The human behavior analysis work described in this paper processes sensor events recorded in eight CASAS smart homes. The smart homes, designed at the Center for Advanced Studies in Adaptive Systems (CASAS), are equipped with passive ambient sensors that provide an indication of the resident’s location within the home. The ambient sensors include passive infrared motion sensors. Several of these are placed on the ceiling in each major room of the house. They are positioned over functional areas, such as the kitchen sink, refrigerator, dining room table, living room couch, office desk, bathroom sink and shower, and the bed. Each of the motion sensor cases also houses an ambient light sensor. Additionally, the homes contain magnetic door sensors. These are placed on all external doors, as well as key cabinets, such as the ones that contain medicine. Each of the magnetic door sensor cases further houses an ambient temperature sensor. All of the sensors are discrete events—they report an “event” when there is a change in state. Figure 2 shows the layout of one example smart home—the one that represents our on-campus testbed.

Table 1 shows a series of sensor messages, or events, that were recorded at the on-campus smart apartment. Each sensor event is a three-tuple containing the message timestamp, the sensor identifier, and the message content. The ambient sensor data that were used for our analysis are available from the CASAS web site at casas.wsu.edu. This study was approved by the Washington State University Institutional Review Board.

### 2.3. Modeling the Smart Home

**Figure 3:**
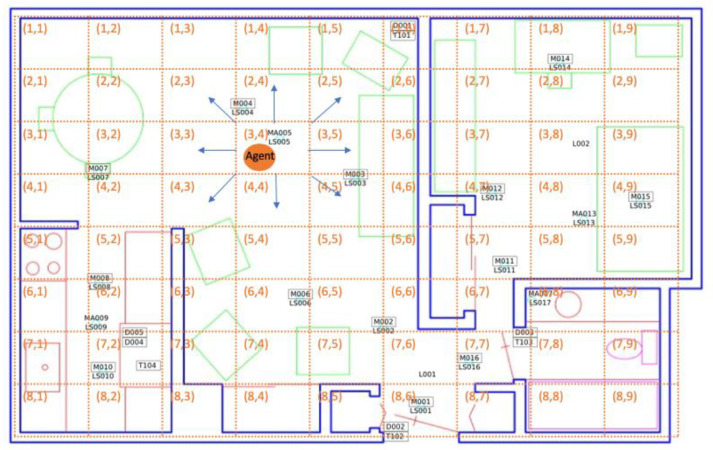
Markov Decision Process (MDP) representation of a smart home resident’s decision-making process in the context of a smart home floorplan.

To model a smart home resident’s decision-making process, we defined an MDP\R with a finite set of states and a finite set of actions. For this problem, each state *s ∈ S* is a spatio-temporal region, specified by a geographical cell location and a time slot. Similarly, action *a ∈ A* is one of nine possible choices. These consist of staying in the same cell or navigating from the current cell to one of the eight adjacent cells. Reward *R* is represented by the inner product of a feature vector *ϕ* and the preference/weight vector *θ*. Figure 3 shows an example of a smart home decision space within the MDP\R. Here, the space is the residence floor plan, represented as a grid containing axis-aligned cells (indicated by dashed lines in the figure). Each cell, labeled with its corresponding row and column number, represents a single state in the MDP. The resident’s navigation choices, or possible actions, are depicted for one cell with blue arrows in the figure.

In Figure 3, the resident (represented as an orange circle) is currently in state s3,4 (i.e., the middle of the living room). Motion sensors are located in positions marked by black rectangles and labeled with the sensor identifier. Sensor identifiers labeled as “Mxxx” are downward-facing passive infrared motion sensors configured to sense a region with a diameter of one meter, “MAxxx” are motion sensors configured to sense an entire room, “LSxxx” are light sensors, and “Dxxx” are motion/temperature sensors. Shapes indicated with green, purple, or pink lines are furniture or appliance items in the apartment. In this example, the resident selects the lower-right movement as the action that will transition the state to s4,5. After executing a sequence of actions, the resident eventually reaches the final state in the observed sequence—s4,9 (near the bed).

#### 2.3.1. Floorplan Quantization

Our algorithm—RRE-IRL—is designed to analyze behavior patterns from ambient sensors embedded in smart homes. As mentioned in Section 2.3, each home is represented as a spatio-temporal grid. Given a home’s floorplan, we can divide the house into equal-sized grid cells and assign a unique identifier to each cell. We can eliminate cells that are unreachable (walls or furniture preventing the resident from moving there). Next, we can divide collected sensor data into daily trajectories, resulting in 365 total trajectories. Each spatio-temporal region *r* is thus a pair containing the grid cell *s* and time *t*. In this way, each resident’s trajectories can be mapped onto sequences of spatio-temporal regions.

In our smart homes, not all grid cells are monitored by sensors. As a result, the residents’ indoor trajectories may not be continuous in the MDP. Consider the home depicted in Figure 2. For one trajectory in this home, motion sensor M007 (located in cell (3,2)) was triggered. The next sensor event was reported by motion sensor M004 (located in cell (2,4)). The path from (3,2) to (2,4) is ambiguous. This uncertainty increases the difficulty of modeling and analyzing indoor trajectories using an MDP.

To resolve this problem, we can impute the missing trajectory steps by finding the cell *x* that has a minimum summed Euclidean distance from the current state to *x* and from *x* to the next observed cell. Only cells that that can be traversed are considered (i.e., no furniture or walls are blocking the path). For our example, cell (2,3) is selected as the next state from cell (3,2) en route to location (2,4). We can repeat this process as needed to form continuous resident trajectories.

#### 2.3.2. Feature Extraction

Individuals make numerous movement-based decisions throughout the day (e.g., when to get out of bed and move to the bathroom, and when to move from the office back to the bedroom at the end of the day). To make such decisions, smart home residents instinctively evaluate multiple factors, or features, related to their current state, *s*. These may include the priority of tasks on their to-do list and their current location in the home (e.g., the current spatio-temporal region, *r*). We designed 14 features—*ϕ=(ϕ_1_,.., ϕ_14_)*—to represent factors that may impact a resident’s decision making. These features are categorized into two groups: The duration that the resident stays at a location (feature names prefixed by “d_”) and the overall activity/movement level at a location (feature names prefixed by “o_”). The feature vector thus contains the set of features listed in Table 2. We assume that trajectory rewards are a linear combination of features and their weights (preference vector). Given observed feature values, we can thus calculate the corresponding preference vector using RRE-IRL, described in the next section.

### 2.4. Relative Entropy IRL

Explicitly learning indoor human movement dynamics is a very challenging problem. Because of the inherent complexity involved in formally modeling the dynamics, we employed a model-free IRL based on Relative Entropy to understand a smart home resident’s behavior based on observed in-home movement trajectories. A smart home resident selects actions based on their own internal policy. If we wanted to learn such a policy, we would attempt to maximize the expected sum of rewards. Alternatively, in the case of an MDP\R where the rewards are unknown, we could determine the optimal policy by maximizing the feature expectation that is extracted from the action sequences (in our case, the resident’s movement trajectories). In our study, our goal was not to learn a policy, but to analyze a person’s routine behavior and determine the differences in behavior between population groups. As a result, we compared resident trajectories. To obtain multiple trajectories from which we could build a model, we considered each day’s worth of sensor events, or movement-based actions, as a separate action sequence to model.

We defined our MDP\R with a finite horizon *h*, implying that the number of time steps to be modeled is finite. In our study, the horizon *h* was a single day, from the beginning of a day (00:00:00) to the end of the same day (23:59:59). We defined a resident’s indoor behavior for one day as a single trajectory, *τ*. Correspondingly, the set of resident daily trajectories is denoted as Τ (τ∈Τ). Let *P(τ)* represent a probability distribution over resident trajectories Τ and *Q(τ)* denote the distribution that is inducted by a baseline policy (e.g., one where *Q(τ)* is a uniform distribution). In this case, Relative Entropy IRL (RelEnt-IRL) minimizes the relative entropy, or Kullback–Leibler divergence [52], between *P(τ)* and *Q(τ)*. This is shown in Equation (5).

(5) $$\begin{array}{c} min_{P}\underset{\tau \in Τ}{\sum }P\left(\tau \right)ln\frac{P\left(\tau \right)}{Q\left(\tau \right)}\\ s.t.\left|\;\underset{\tau \in Τ}{\sum }P\left(\tau |\theta \right)\cdot \phi_{i}^{\tau }-\mu_{i}\left(\tau \right)\right|\leq \varepsilon_{i},\forall i\in 1,..,k\\ \underset{\tau \in Τ}{\sum }P\left(\tau |\theta \right)=1\\ P(\tau |\theta )\geq 0 \end{array}$$

In Equation (5), *k* is the number of features, $\phi_{i}^{\tau }$ is the *i*th feature in vector $\phi^{\tau }$ that is extracted from trajectory *τ*, $\mu_{i}\left(\tau \right)$ is the *i*th feature expectation in *μ(τ)*, and $\varepsilon_{i}$ is a threshold based on Hoeffding’s bound [41]. This constrained optimization problem can be solved in two steps: By introducing Lagrangian multipliers *L* and solving the Lagrange dual function *g*. Using this approach, *P* can be defined as a function of *τ* and *θ*, as shown in Equation (6):

(6) $$P\left(\tau |\theta \right)=\frac{Q\left(\tau \right)\exp\left(\theta \cdot \phi^{\tau }\right)}{\sum_{\tau \in Τ}Q\left(\tau \right)\exp\left(\theta \cdot \phi^{\tau }\right)}$$

$$=\frac{1}{Z\left(\theta \right)}Q\left(\tau \right)\exp\left(\theta \cdot \phi^{\tau }\right),\forall \tau \in T,$$

where *Z(θ) =* $Q\left(\tau \right)\exp\left(\theta \cdot \phi^{\tau }\right)$. The corresponding dual function is shown in Equation (7):

(7) g(θ)=θ·μ=lnZ(θ)−|θ|·ε,

where *|θ|* is the absolute value of each element in the weight vector *|θ| = (|θ*_1_*|,..., |θ*_k_*|),* $\varepsilon =\left(\varepsilon_{1},..,\varepsilon_{k}\right)$ is a vector of $\varepsilon_{i}$, and *k* is the number of features. The gradient of the dual function is shown in Equation (8):

(8) $$\frac{\partial }{\partial \theta_{i}}g\left(\theta \right)=\mu_{i}-\underset{\tau \in Τ}{\sum }P\left(\tau |\theta \right)\cdot \phi_{i}^{\tau }-\alpha_{i}\cdot \varepsilon_{i},$$

where $\alpha_{i}=1$ if $\theta_{i}\geq 1$; otherwise, $\alpha_{i}=-1$.

To efficiently approximate the gradient in Equation (8), an importance sampling method is used for a set of *N* trajectories, $T_{N}^{\tau }$, while executing policy *π*. The term $\underset{\tau \in Τ}{\sum }P\left(\tau |\theta \right)\cdot \phi_{i}^{\tau }$ can be estimated for any $\tau \in T_{N}^{\tau }$. We can assume that *Q(τ)*, representing the distribution of trajectories from a base policy in Equation (5), can be decomposed into the expression $Q\left(\tau \right)=S_{tran}\left(\tau \right)\cdot A_{tran}\left(\tau \right),$ where $S_{tran}\left(\tau \right)=D\left(s_{0}\right)\prod_{i=1}^{H}T\left(s_{i},a_{i},s_{i+1}\right)$ is the joint probability of the state transitions in a trajectory *τ* given the initial state distribution $D\left(s_{0}\right)$ and $A_{tran}\left(\tau \right)$ is the joint probability of the actions executed on states in *τ*. Based on this formulation, the sample-based gradient can be approximated as shown in Equation (9):

(9) $$\frac{\partial }{\partial \theta_{i}}g\left(\theta \right)=\mu_{i}-\underset{\tau \in Τ}{\sum }P\left(\tau |\theta \right)\cdot \phi_{i}^{\tau }-\alpha_{i}\cdot \varepsilon_{i}.$$

Algorithm 1—Resident Relative Entropy IRL (RRE-IRL)—summarizes the Relative Entropy IRL procedure for determining a smart home resident’s reward function (preference/weight vector) based on observed indoor movement trajectories.

**Algorithm 1:** Resident Relative Entropy IRL
**input:**
set of trajectories ***T***
set of sample trajectories ***T****_N_**(T**_N_⊂**T**)*
policy *π* approximated by ***T****_N_*
threshold vector ε
learning rate vector α
*N×k* feature matrix *Ф* # N=number of trajectories, k=number of features
**output:**
preference/weight vector *θ*
**initialize:**
weight vector *θ* with random numbers and feature expectation *μ*


**while**
($\frac{\partial }{\partial \theta_{i}}g\left(\theta \right)>\varepsilon_{i}$) **do**
calculate $\frac{\partial }{\partial \theta_{i}}g\left(\theta \right)$ using Equation (9)
update $\theta_{i}=\theta_{i}+\alpha_{i}\cdot \frac{\partial }{\partial \theta_{i}}g\left(\theta \right)$
**end**




**return**
θ

## 3. Results

Once we had defined the Resident Relative Entropy IRL algorithm (RRE-IRL), we used this algorithm to quantify and characterize differences in behavior patterns. In particular, we performed the following experiments:

- Experiment 1: Analyze and compare smart home behavior patterns for a single resident at two points in time. Determining whether the learned preference/weight vectors are significantly different gives us an indication of whether a person’s behavior is changing over time due to influences such as seasonal changes, changes in the environment, or changes in health;
- Experiment 2: Quantify change in smart home behavior patterns for multiple smart home residents within the same diagnosis group. We hypothesized that the amount of change we would observe in the behavior patterns, as defined by the learned preference/weight vectors, would be greater between different individuals than for one individual at different time points. We hypothesized that this would be particularly true when multiple individuals were drawn from the same health diagnosis sub-population;
- Experiment 3: Quantify change in smart home behavior patterns for multiple smart home residents from different diagnosis groups. We hypothesized that the amount of change we would observe in behavior patterns would be greater between individuals from different diagnosis groups than for either Experiment 1 or Experiment (2);
- Experiment 4: Characterize the nature of behavioral change that is observed between smart home residents from different diagnosis groups. We analyzed the preference/weight vectors that were learned for different smart home residents to determine the nature of the change that was observed between individuals who were healthy and those who were experiencing cognitive decline. We also used the preference vectors to predict the diagnosis group for an individual smart home resident.

### 3.1. Experimental Conditions

**Figure 4:**
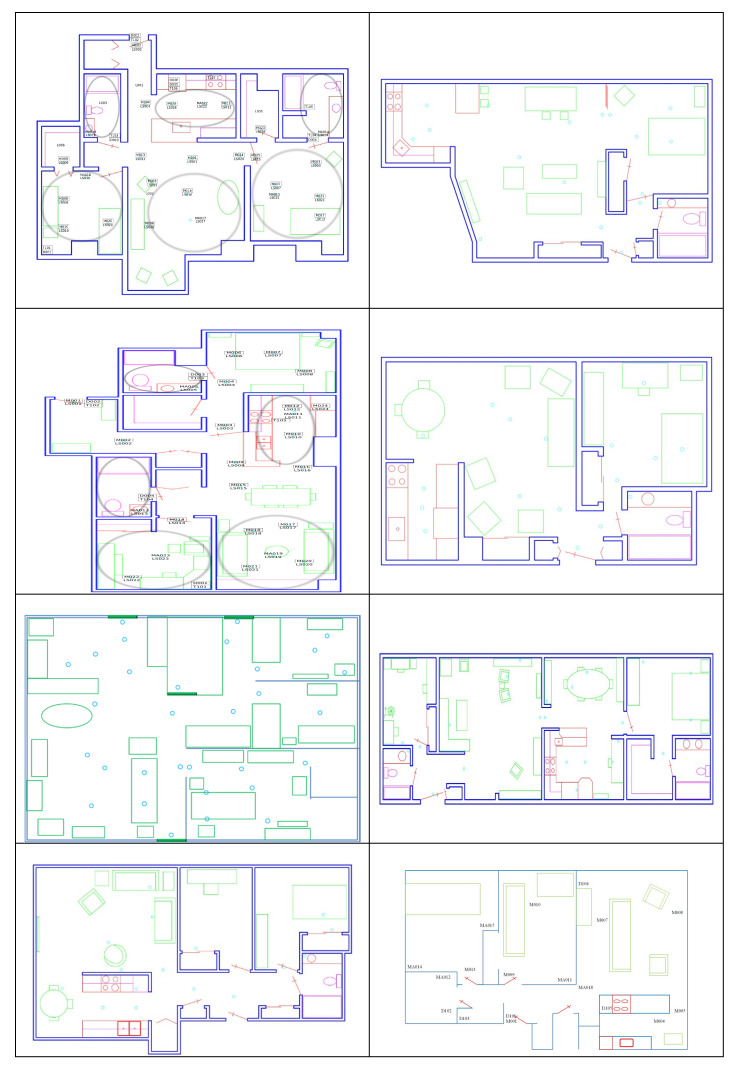
Smart home floorplans. Left column from top to bottom: Home 1, Home 2, Home 3, and Home 4. Right column from top to bottom: Home 5, Home 6, Home 7, and Home 8.

We analyzed movement-based behavior data collected in eight smart homes. To facilitate a comparative analysis of population sub-groups, we selected four homes with older adult residents who had been diagnosed as cognitively healthy and four homes with older adult residents who were experiencing cognitive decline. A summary of the eight homes is provided in Table 3. Floorplans for the corresponding homes are shown in Figure 4.

### 3.2. Within-Home Analysis

We began by analyzing data within each home separately. By examining the learned preference vectors, we can observe how a smart home resident behaves on a regular basis. Based on the features that we designed, we analyzed how long they spent at locations throughout the house and how active they were at the locations. Different feature specifications would have allowed us to analyze alternative aspects of resident behavior. For example, in future work, we can introduce a feature such as the walking speed, quantified as the normalized rate of moving from one region of the home to another. Applying RRE-IRL to such a feature would allow us to assess the relationship between cognitive health and the walking gait. Researchers have indicated that brain health and walking speed often decline together [53]. They have provided evidence for this hypothesis by performing clinical cognitive assessment, together with scoring a scripted gait speed task. In contrast, the type of analysis we propose can provide an ecologically-valid method of validating this hypothesis.

**Figure 5:**
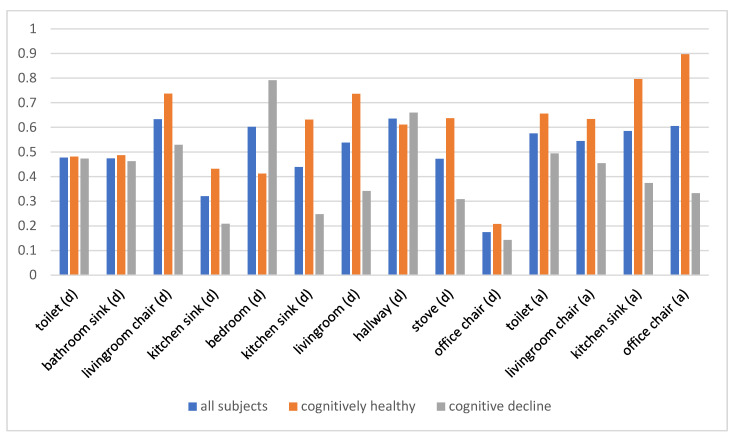
Weights of location-based duration (d) and activity level (a) features learned using the RRE-IRL algorithm.

Table 4 summarizes the feature vectors for the eight analyzed homes. As Liu et al. suggest, the preference values (i.e., reward weights) can be interpreted as the importance of features to the corresponding individual [54]. The preference values for all subjects, both those who were cognitively healthy and those with cognitive decline, are shown in Figure 5. Overall, the subjects, all of whom are older adults, show a preference for time spent in a favorite living room chair and in the hallway connecting regions of the home. Time spent in the office chair or at the kitchen sink has much less of an influence on their routine. The two population subgroups—cognitively healthy and cognitive decline groups—differ most greatly in terms of time spent in the bedroom, at the kitchen sink and stove, and in the living room. Subjects with cognitive decline showed a stronger preference for time in the bedroom, while cognitively healthy subjects exhibited preference for time in the living room and kitchen. Additionally, cognitively healthy subjects showed a stronger preference for overall activity in the monitored areas.

Additionally, we analyzed the impact of time on behavioral changes within each home; that is, given the behavior preference vector of a smart home resident for the first two months of data collection (e.g., $\theta_{Home1}^{1,2}$), we compared the vector with a vector learned from the same home over a second month-long time period (e.g., compared $\theta_{Home1}^{1,2}$ with $\theta_{Home1}^{n-1,n}$). We wanted to quantify the amount of behavioral change that occurs for a single person over time and determine if the change is statistically significant. To quantify change within a home over time, we applied a paired t-test to the individual-day preference values for the time frames. We repeated this computation for each of the individual features in the preference vector. 

The results are summarized in Table 5 for the duration features, because these resulted in lower probabilities. We also report the per-home mean probability over all features and over just the duration features. The mean overall probability for homes with residents experiencing cognitive decline is 0.40 and for cognitively healthy residents is 0.43. Considering only duration features, the mean probability for residents with cognitive decline is 0.35 and the mean probability for cognitively healthy residents is 0.45.

We note that the probability that the samples collected at different time points belong to the same distribution is lower for individuals experiencing cognitive decline than for cognitively healthy participants. The observation holds for duration features, as well as for the entire feature vector. This is consistent with the literature, which indicates that day-to-day variability in behavior is an indicator of a change in cognitive health [55]. One smart home resident in particular, the resident living in Home 2, exhibited changes in behavior that were statistically significant. These changes were reflected in the time that the resident spent in the bedroom, kitchen, living room, and hallway. The change is more dramatic given the fact that Home 2 was one of the shorter data collection periods. In contrast, Home 5, with the longest data collection period, exhibited a very small amount of change in preference vectors from the beginning to end of data collection.

We further note that there are few changes that are statistically significant. There are several possible explanations for this. First, not all individuals actually dramatically change their behavior over time. Particularly for older adults, behavior becomes very structured and many do not change much, even as they experience changes in their health status. Second, some changes in behavior may not be reflected in coarse-granularity ambient sensor data. For example, cognitive tasks may take more effort for individuals experiencing cognitive decline, but these changes may not result in substantially-different movement trajectories. Third, a limitation of this study is the relatively small sample size. Future work may expand the number of homes considered and address this current limitation.

### 3.3. Between-Person Analysis Within the Same Diagnosis Group

Next, we are interested in quantifying behavioral differences for multiple individuals within the same diagnosis group. We hypothesized that between-person differences would be greater (correspondingly, the p values would be smaller) for between-person differences than single-person between-time differences. We quantified the differences by performing an ANOVA calculation over the learned preference vectors for each month within the analyzed smart homes.

We can make several observations based on the ANOVA results summarized in Table 6. First, the overall differences between individuals are greater (the p values are smaller) for the cognitively healthy participants than for those with cognitive decline. Second, the differences between individuals experiencing cognitive decline are comparable to over-time differences for individuals within this diagnosis category. Since most of these participants transitioned to greater cognitive impairment over time, this result is not surprising. Third, the between-person differences for cognitively healthy participants are greater than the within-person, over-time differences. Specifically, we can see that this group differs significantly (*p* < 0.05) in terms of time spent in the living room, as well as the kitchen sink and the office chair. These differences may reflect lifestyle differences, balancing time spent cooking, working, and relaxing. For the participants experiencing cognitive decline, the greatest difference occurs in time spent at the toilet, which exhibits a statistically significant difference.

Similar to the within-home analysis results, not every difference between homes is statistically significant. The impact of the sample size on these results can be explored further as part of our future work. Additionally, finer-grained sensors (e.g., wearable accelerometers) can be integrated into the data collection to increase the sensitivity of behavior monitoring for features that may be impacted by the health status, such as gait characteristics.

### 3.4. Between-Group Analysis

Finally, we investigated the differences between diagnosis groups. Here, we expected that the differences would be large (small p values). We hypothesized that the differences would be greater than for the within-home comparison or the between-person, within-group comparison. As before, we quantified differences by performing an ANOVA calculation over the learned preference vectors for each month. In this experiment, we aggregated data from the four homes whose residents were experiencing cognitive decline into one group. Similarly, we aggregated data from the four homes with cognitively healthy participants into one group. The results are summarized in Table 7.

As can be seen from the results, the overall mean is smaller than all previous experiments, except for between-person differences for the cognitively healthy group. This table also shows the greatest number of features that exhibit a statistically significant difference, including time spent at the living room chair (and the general living room area), kitchen sink, and office chair.

### 3.5. Characterizing Behavioral Change for Automated Health Assessment

Based on the results in the previous section, we know that significant differences exist between preference vectors, and thus behavior patterns, for different health diagnosis groups. We therefore hypothesized that we can predict a person’s diagnosis group based solely on these learned preference vectors. To validate this hypothesis, we performed a leave-one-out classification experiment using a random forest model with 100 trees, each formed using the entropy measure. The results are shown in Table 8 and indicate that the preference vectors do provide a basis for predicting a cognitive health diagnosis from sensor-observed longitudinal behavior.

### 3.6. Determining Behavior Indicators that Distinguish Population Subgroups

**Figure 6:**
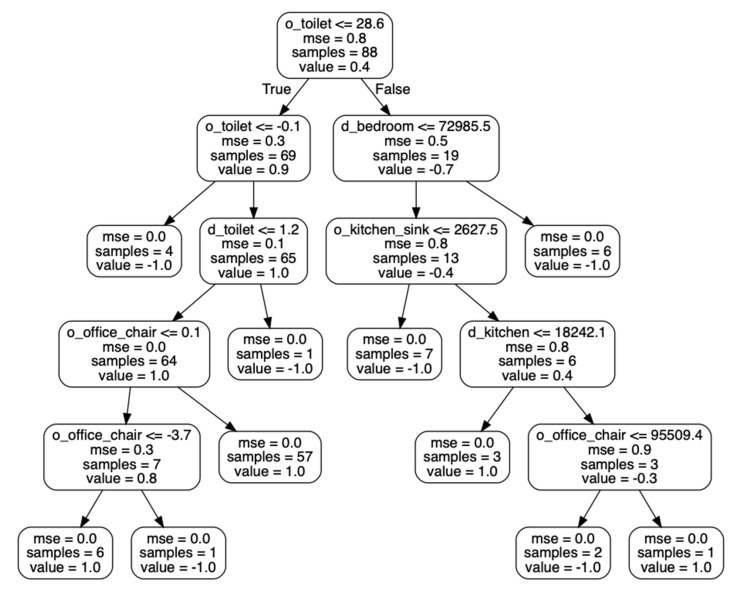
One of the learned decision trees (based on non-normalized values) used to predict whether a smart home resident is cognitively healthy (value = 1.0) or is experiencing cognitive decline (value = −1.0).

When a classification algorithm is applied to learned preference vectors to distinguish health diagnosis groups, the results can also provide insights on specific behaviors that are consistent with different health statuses. As an example, Figure 6 shows one of the decision trees that was learned from our dataset to distinguish cognitively healthy smart home residents from residents who are experiencing cognitive decline. The tree indicates that the activity level near the toilet is a primary indicator of cognitive health in this dataset (an observation that is consistent with our earlier experiments). Other indicators are the activity level (movement level) near the office chair and amount of time spent near the toilet and in the bedroom and kitchen.

Additionally, we performed feature-importance selection from the training data. The results, summarized in Table 9, are consistent with the preference vectors summarized in Table 4. Specifically, the time spent near the office chair and near the kitchen sink has little influence on the overall routine, while areas such as the bedroom, bathroom, and living room exhibit a greater influence. All of the participants in this study were over the age of 65. These preferences may be consistent with this demographic. In future work, we would like to analyze data for a greater age range, which may highlight stronger behavioral influences for time in the office chair working and at the kitchen sink cooking or washing dishes. Two of the largest distinctions between the two health groups are the activity level near the toilet and amount of time spent near the toilet. Some of the individuals in the cognitive decline group also dealt with additional health and mobility challenges that may have resulted in a greater bathroom time and more work required to get to and from the bathroom, particularly in the middle of the night. The findings in this work may help clinicians and engineers to improve assessment measures of health based on behavior. Automating such assessment assists with designing treatments and extending functional independence.

## 4. Discussion and Conclusions

By analyzing ambient sensor data using our proposed RRE-IRL algorithm, we were able to extract preference vectors that indicate and quantify aspects of a person’s routine behavior. By using these tools, we were able to compare changes in behavioral norms over time. We also compared differences in behavior for individuals in the same health diagnosis group and across groups. We found that changes in behavior occurred over time for all of the study participants. The probability that behavior preferences remained the same (were drawn from the same distribution) was lower than 0.55 for all eight of the smart home residents. The probability was lower for residents experiencing cognitive decline (0.40) than for cognitively healthy residents (0.43). This difference may be due to the ways in which residents adapt to their changing health status, such as using memory-compensatory behavior (e.g., reminder notes) or new behavior that may accompany cognitive decline (e.g., perseveration and wandering).

We also found that behavior was more varied between individuals in the cognitively healthy group (probability that observations are drawn from the same distribution is differences in behavior) were quantitatively larger between residents in the cognitively healthy group (probability that behavior preferences are drawn from the same distribution is 0.32) than the cognitive decline group (probability is 0.42). The difference between health groups was also quantified. When comparing aggregated behavior between groups of residents from the two diagnosis groups, the probability that the observed behavior was drawn from the same distribution was 0.32. These measures provide insight on behavior patterns. Changes in these measures also help us quantify the extent of behavioral change that occurs over time, between different people, and between diagnosis groups.

By feeding these measures, or behavior preferences, into a classification algorithm, we offer a basis for automating the detection of cognitive health decline. For these eight smart homes, a random forest classifier was able to predict the health diagnosis group with an accuracy of 0.84. 

This work introduces a tool for quantifying and assessing observed behavior for an indoor environment. While the data did support a comparison of behavior between health diagnosis groups, there are limitations of the current analysis. One limitation is the participant sample size. Our analyses were based on a large set of data collected over many days from actual smart homes. However, data for only eight participants were considered. Collecting and analyzing data from a larger population of individuals with different health statuses may allow us to generate additional findings and yield more robust health prediction results.

A second limitation is the coarse granularity of the information that is provided by ambient sensors. These sensors provide information on resident navigation patterns within homes. As a result, the captured features also indicate movement patterns, such as the time spent in regions of the home and activity level in those regions. Including data from other types of sensors can increase the diversity of information that we analyze. For example, wearable sensors may provide insights on a person’s gait that are useful for detecting changes in their health status. In future work, we will investigate methods for predicting health change based on changes in a person’s desiderata. The results may provide timely and informed interventions to prevent and help with a variety of health challenges.

## Figures and Tables

**Table 1:** An example of sensor messages recorded from the on-campus smart home testbed. Each sensor message contains the message date/time, the sensor ID, and the sensor message.

Timestamp	Sensor ID	Message
02/06/2009 17:52:28	M025	ON
02/06/2009 17:52:32	M025	OFF
02/06/2009 17:52:35	M025	ON
02/06/2009 17:52:36	M025	OFF
02/06/2009 17:52:37	M045	ON
02/06/2009 17:52:38	M025	ON
02/06/2009 17:52:44	M045	OFF
02/06/2009 17:53:31	M024	ON
02/06/2009 17:53:32	M019	ON
02/06/2009 17:53:33	M021	ON
02/06/2009 17:53:33	M025	OFF
02/06/2009 17:53:34	M021	OFF
02/06/2009 17:53:34	M018	ON
02/06/2009 17:53:36	M051	ON
02/06/2009 17:53:36	M024	OFF

**Table 2:** Set of features guiding smart home movement decisions.

d_Toilet	d_Bathroom_Sink	d_Livingroom_Chair	d_Kitchen_Sink
d_bedroom	d_kitchen	d_livingroom	d_hallway
d_stove	d_office_chair	o_toilet	o_livingroom_chair
o_kitchen_sink	o_office_chair		

**Table 3:** Summary of information for eight smart home residents.

Group	ID	Health Diagnosis	#Sensors	Duration of Data Collection	Number of Month-Long Samples	Total Number of Sensor Events
Cognitive decline	Home 1	Mild Cognitive Impairment (MCI)	21 downward-facing motion (motion); 2 motion area (ma)	843 days	26	4,785,969
	Home 2	MCI	19 motion; 2 ma	223 days	7	876,303
	Home 3	MCI	26 motion; 0 ma	682 days	22	5,167,574
	Home 4	MCI, early dementia	11 motion; 2 ma	149 days	5	24,948
Cognitively healthy	Home 5	Healthy	13 motion; 1 temperature	1788 days	56	5,761,601
	Home 6	Healthy	13 motion	1591 days	49	4,850,970
	Home 7	Healthy	18 motion; 2 ma	379 days	12	2,292,312
	Home 8	Healthy	10 motion; 1 ma	969 days	31	1,853,637

**Table 4:** Smart home normalized preference vectors.

Home ID	d_Toilet	d_Bath-Room Sink	d_Livingroom Chair	d_Kitchen Sink	d_Bedroom	d_Kitchen	d_Living-Room
1	0.631	0.631	0.224	0.000	1.000	0.073	0.096
2	0.059	0.061	0.057	0.000	0.847	0.445	0.047
3	0.377	0.379	1.000	0.000	0.319	0.133	0.892
4	0.824	0.777	0.836	0.836	1.000	0.340	0.329
5	0.382	0.405	0.435	0.407	0.615	0.603	0.429
6	0.988	0.988	0.998	1.000	0.292	0.995	0.999
7	0.308	0.308	0.745	0.318	0.000	0.379	0.745
8	0.246	0.246	0.770	0.000	0.743	0.545	0.770
**Home ID**	**d_Hallway**	**d_Stove**	**d_Office Chair**	**o_Toilet**	**o_Living-Room Chair**	**o_Kitchen Sink**	**o_Office Chair**
1	0.302	0.326	0.401	0.666	0.262	0.292	0.281
2	1.000	0.049	0.048	0.060	0.047	0.000	0.046
3	0.467	0.001	0.123	0.524	0.711	0.486	0.313
4	0.871	0.855	0.000	0.725	0.797	0.719	0.689
5	0.917	0.661	0.000	0.381	0.539	1.000	0.854
6	0.993	0.992	0.000	0.996	0.990	0.993	0.767
7	0.068	0.318	0.252	1.000	0.594	0.652	0.893
8	0.464	0.578	0.580	0.246	0.413	0.538	1.000

**Table 5:** Paired *t*-test results for within-home behavior comparisons. (* = Result is statistically significant (*p* < 0.05).).

ID	d_Toilet	d_Bath-Room Sink	d_Living-Room Chair	d_Kit-Chen Sink	d_Bed-Room	d_Kit-Chen	d_Living-Room	d_Hall-way	d_Stove	d_Office-Chair	Dura-tion Mean	Overall Mean
1	0.39	0.39	0.10	0.35	0.07	0.30	0.35	0.40	0.25	0.96	0.36	0.40
2	0.45	0.38	0.33	0.27	0.04 *	0.02 *	0.03 *	0.02 *	0.29	0.07	0.19	0.29
3	0.74	0.74	0.37	0.55	0.46	0.47	0.54	0.47	0.55	0.48	0.54	0.54
4	0.46	0.40	0.32	0.28	0.35	0.09	0.84	0.12	0.30	0.09	0.33	0.39
5	0.42	0.87	0.28	0.56	0.54	0.65	0.28	0.81	0.73	0.37	0.55	0.51
6	0.56	0.03 *	0.08	0.58	0.92	0.16	0.83	0.09	0.55	0.29	0.41	0.46
7	0.14	0.14	0.31	0.49	0.17	0.55	0.31	0.15	0.49	0.76	0.35	0.33
8	0.91	0.22	0.06	0.78	0.57	0.61	0.06	0.66	0.66	0.49	0.50	0.44

**Table 6:** One-way ANOVA results for between-home behavior comparisons within the same diagnosis group. Entries in blue with a standard font indicate the duration in a location, and entries in green with an italic font indicate the activity level in a location. (* = Result is statistically significant (*p* < 0.05).).

**Cognitive** **Decline**	**d_Toilet**	**d_Bath-Room Sink**	**d_Living-Room Chair**	**d_Kitchen Sink**	**d_Bed-Room**	**d_Kitchen**	**d_Living-Room**	**d_Hall-way**
	0.29	0.29	0.29	0.24	0.67	0.56	0.79	0.62
	**d_Stove**	**d_Office Chair**	**o_Toilet**	**o_Living-Room Chair**	**o_Kitchen Sink**	**o_Office Chair**	**Overall Mean**	
	0.85	0.78	0.00 *	0.12	0.10	0.35	0.42	
**Cognitively Healthy**	**d_Toilet**	**d_Bath-Room Sink**	**d_Living-Room Chair**	**d_Kitchen Sink**	**d_Bed-Room**	**d_Kitchen**	**d_Living-Room**	**d_Hall-way**
	0.10	0.10	0.02 *	0.62	0.99	0.62	0.02 *	0.58
	**d_Stove**	**d_Office Chair**	**o_Toilet**	**o_Living-Room Chair**	**o_Kitchen Sink**	**o_Office Chair**	**Overall Mean**	
	0.58	0.95	0.17	0.26	0.00 *	0.00 *	0.32	

**Table 7:** One-way ANOVA results for between-group behavior comparisons. Entries in blue indicate the duration in a location, and entries in green indicate the activity level in a location. (* = Result is statistically significant (*p* < 0.05).).

**d_Toilet**	**d_Bathroom Sink**	**d_Livingroom Chair**	**d_Kitchen Sink**	**d_Bedroom**	**d_Kitchen**	**d_Living-Room**	**d_Hallway**
0.10	0.10	0.02 *	0.62	0.99	0.62	0.02 *	0.58
**d_Stove**	**d_Office Chair**	**o_Toilet**	**o_Livingroom Chair**	**o_Kitchen Sink**	**o_Office Chair**	**Overall Mean**	
0.95	0.17	0.26	0.00 *	0.00 *	0.01 *	0.32	

**Table 8:** Health diagnosis prediction results. Here, cognitive decline represents the positive class.

Accuracy	Precision	Recall	F1 Score
0.84	0.88	0.90	0.89

**Table 9:** Decision tree-based importance for each feature in distinguishing cognitively healthy and cognitive decline subjects based on behavior routines.

o_Toilet	d_Toilet	d_Hallway	d_Livingroom	o_Office Chair	d_Bathroom Sink	o_Living-Room Chair
0.33	0.22	0.10	0.06	0.06	0.04	0.04
**d_Living-Room Chair**	**d_Bedroom**	**d_Kitchen**	**d_Stove**	**d_Office Chair**	**o_Kitchen Sink**	**d_Kitchen Sink**
0.03	0.02	0.02	0.02	0.02	0.02	0.01
